# Electrical and Thermal Conductivity of Polylactic Acid (PLA)-Based Biocomposites by Incorporation of Nano-Graphite Fabricated with Fused Deposition Modeling

**DOI:** 10.3390/polym11030549

**Published:** 2019-03-22

**Authors:** Rui Guo, Zechun Ren, Hongjie Bi, Min Xu, Liping Cai

**Affiliations:** 1 Key Laboratory of Bio-based Material Science and Technology (Ministry of Education), Material Science and Engineering College, Northeast Forestry University, Harbin 150040, China; guorui0527@163.com (R.G.); yourong_rzc@163.com (Z.R.); bihongjie1016@163.com (H.B.); 2 Mechanical and Energy Engineering Department, University of North Texas, Denton, TX 76201, USA; Liping.Cai@unt.edu; 3 College of Materials Science and Engineering, Nanjing Forestry University, Nanjing 210037, China

**Keywords:** polylactic acid, biocomposites, nano-graphite, electrical conductivity, fused deposition modeling, tannic acid-functionalized graphite

## Abstract

The aim of the study was to improve the electrical and thermal conductivity of the polylactic acid/wood flour/thermoplastic polyurethane composites by Fused Deposition Modeling (FDM). The results showed that, when the addition amount of nano-graphite reached 25 pbw, the volume resistivity of the composites decreased to 10^8^ Ω·m, which was a significant reduction, indicating that the conductive network was already formed. It also had good thermal conductivity, mechanical properties, and thermal stability. The adding of the redox graphene (rGO) combined with graphite into the composites, compared to the tannic acid-functionalized graphite or the multi-walled carbon nanotubes, can be an effective method to improve the performance of the biocomposites, because the resistivity reduced by one order magnitude and the thermal conductivity increased by 25.71%. Models printed by FDM illustrated that the composite filaments have a certain flexibility and can be printed onto paper or flexible baseplates.

## 1. Introduction

Polymer-based composites filled with carbon nanofillers, such as nano-graphite, carbon nanotubes, and graphene, have many potential applications, such as thermal control devices and electronic parts. With the increasing demand for electronic devices, the development of polymer-based composites with high thermal, electrical conductivity and low cost has become a research hotspot [1,2]. The carbon nano-filler can impart certain electrical and thermal conductivity of the composites while reinforcing the polymer [3,4,5]. At the same time there were some drawbacks during their applications, e.g., carbon nano-fillers could not disperse in polymers and had a relatively high cost [6,7,8]. Among the carbon nano-fillers, nano-graphite is composed of stacked two-dimensional graphene sheets, which is an ideal nano-filler to enhance polymer matrix due to its good aspect ratio, unique two-dimensional structure, and low manufacturing cost [9].

In addition to the consideration of environmental issues and resource shortages, it is important to choose an applicable polymer as the matrix of the composites. As a biodegradable and environment-friendly bioplastic [10,11], polylactic acid (PLA) can be produced by corns and other food crops [12,13,14] and has been widely used as matrixes for Fused Deposition Modeling (FDM) technology recently [15,16]. However, the brittleness and high cost of PLA limits its application [17]. Therefore, the addition of natural fibers, which are also biodegradable [18,19] for example, the adding of wood flour (WF) into PLA, may be an efficient way to reduce the cost and reinforce the PLA [20,21]. Patanwala et al. used carbon nanotubes and PLA to prepare FDM filaments by melt extrusion. When 5 wt% of carbon nanotubes was added, the Young’s modulus of the composite was improved, but the tensile strength decreased [22]. Daniel et al. compounded PLA with carbon black and graphene respectively using the FDM technology. After the filament was extruded by FDM, its resistivity decreased compared with that before the extrusion [23]. Yu et al. introduced graphene or carbon nanotubes into PLA and obtained filaments suitable for FDM by multiple melt extrusion. The incorporation of graphene or carbon nanotubes significantly reduced the electrical conductivity of the PLA-based composites [24].

The objective of this research is to investigate the effect of incorporating carbon nano-filler on the electrical and thermal conductivity of the PLA/WF composites fabricated by FDM technology, especially the nano-graphite. Nano-graphite is cost-effective, but there are few reports on nano-graphite reinforced PLA for FDM technology. Thermoplastic Polyurethane (TPU) is used to improve the toughness of the PLA/WF composites according to our previous study [25]. With high tensile modulus, TPU is a linear multi-block copolymer with hard and soft chain segments [26], and suitable for melt blending [27]. The tannic acid (TA)-functionalized nano-graphite was made and added into the composites to improve the interfacial compatibility and the thermal conductivity of the composites. Surface modification by covalent or non-covalent functionalization is an effective method to improve dispersion and interfacial adhesion of nano-fillers in the matrix [28,29,30,31]. Covalent functionalization usually destroys the conduction of the sp^2−^ hybridized network that is required for good thermal conductivity, thereby reducing the thermal conductivity of the nano-fillers. However, the non-covalent functionalization involving weak interaction was considered as the optimal choice to improve the interfacial properties of the nano-fillers because it can maintain the high thermal conductivity of the fillers [1,32,33,34,35]. The phenolic groups attached to tannic acid can endow the nano-filler with high chemical and physical bonding ability through van der Waals forces and hydrogen bonding. Multi-walled carbon nanotubes (MCNT) and redox graphene (rGO) were used to synergize with graphite in order to improve the electrical conductivity of the composites.

## 2. Materials and Methods

### 2.1. Materials

Nano-graphite (a diameter of 1.5–2.0 μm, specific gravity of 0.10–0.15 g·cm^−3^) and rGO (average diameter of 40 μm, thickness of 2.50–5.50 nm) were purchased from Qingdao Tianyuanda Graphite Co., Ltd. (Qingdao, China). Multi-walled carbon nanotubes (XFM22) were obtained from XFNano Material Technology Co., Ltd. (Nanjing, China), with an out diameter of 20–30 nm, length of 0.5–2 µm, and purity >95%, of which the number of walls is 23–25, and the aspect ratio is less than 100. PLA (4032D) and Thermoplastic Polyurethane (TPU, 85A) were supplied by NatureWorks Co. Ltd. (Blair, NE, USA) and BASF Co. (Ludwigshafen, Germany), respectively. Poplar wood flour used in the study was 100 meshes. Supplied by Shandong Qilu Petrochemical Engineering Co., Ltd. Zibo, China, polyethylene wax (PEWax) was usually used as a lubricant. Tannic acid (TA) was purchased from Tianjin Fushen Chemical Reagent Co., Ltd. (Tianjin, China).

### 2.2. Sample Preparation

**Figure 1:**
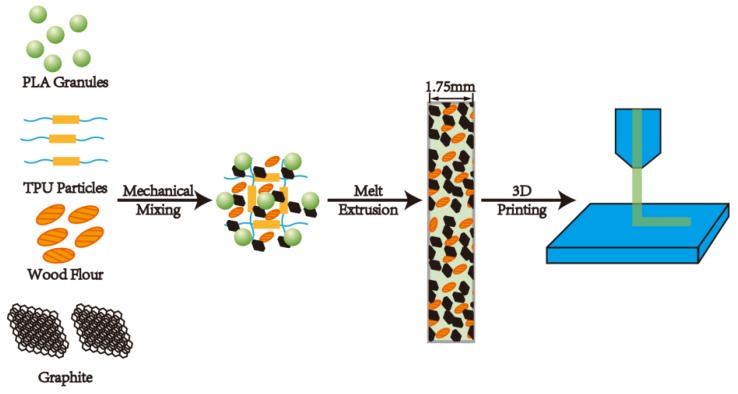
A schematic diagram of the composites preparation process.

PLA was dried at 50 °C for 8 h, nano-graphite (G) was dried at 80 °C for 12 h, and both WF and TPU were dried at 103 °C for 12 h before processing. The formulation of the composites is shown in Table 1. The raw materials were weighed according to Table 1, then were fed into a co-rotating twin-screw extruder (Nanjing Rubber and Plastic Machinery Factory, Nanjing, China, SJSH-30) with a screw L/D of 30:1 to obtain granules. The screw speed was set at 40 rpm, and the temperature from the inlet zone to the outlet zone was 160, 170, 180, 190, 190, 160, and 155 °C, respectively. In order to make the granules into filaments with a diameter of 1.75 mm for application to the FDM, the granules were sent to a single-screw extruder (Dongguan Songhu Plastic Machine Co., Ltd., Dongguan, China, SHSJ25), with a screw L/D of 12:1 and a die temperature of 180 °C. The filaments were extruded by the extruder with a die diameter of 3.5 mm, stretched to a diameter of 1.75 mm ± 0.1 mm under the action of traction, and cooled by water at room temperature. Lastly, the samples were printed by a FDM printer. A schematic diagram of the composite preparation process is shown in Figure 1.

The optimum graphite content, at which the composites could gain relatively good mechanical properties and conductivities, was determined by the experiment in Table 1, and then the composite was modified according to Table 2. TA and nano-graphite were added to the solution of dimethylformamide (DMF) in a mass ratio of 1:4. The solution was magnetically stirred for 10 min, then ultrasonically dispersed using a sonicator (Ningbo Xinzhi Biotechnology Co., Ltd., Ningbo, China) with a pause model of 400 W for 1 h, and finally stirred at room temperature for 4 h. The resulted suspension was filtered, washed three times with deionized water, and dried at 80 °C for 12 h to obtain TA-G. The mass ratios of G/TA-G, G/rGO, and G/MCNT added into the composites were 1:1, 3:1, and 3:1, respectively. The MCNT used in the experiment was not functionalized.

The printing parameters are listed below. The nozzle size of the FDM printer was 1 mm, and the layer thickness of the samples was 0.4 mm. The printing speed was 15 mm/s. The temperature of the nozzle and print bed were 210 °C and 40 °C, respectively. The infill density was 100% and the infill pattern was rectilinear.

### 2.3. Characterization

#### 2.3.1. Electrical Resistivity

The volume electrical resistivity of the composites was tested by a ZC36 high resistance meter (Shanghai Yanggao Electric Appliance Co., Ltd., Shanghai, China). The sample size was 90 × 90 × 1.6 mm^3^ (L × W × H). The electrical resistivity (*ρ*_v_) of the composites was calculated via Equation (1).

(1) $$\rho_{v}\left(\Omega \cdot m\right)=R_{v}\times \frac{\pi \times {\left(D_{1}+g\right)}^{2}}{4\times h}$$

where, *D*_1_ is the diameter of the measuring electrode (equal to 50 mm), *g* is the gap between the measuring electrode and the guard electrode (equal to 2 mm), and *h* is the thickness of the tested sample.

#### 2.3.2. Thermal Conductivity

Thermal conductivity was measured by the nanoflash diffusivity instrument (Netzsch LFA447, Bavaria, Germany). The samples with a diameter of 12.60–12.70 mm were tested. The thermal conductivity (λ) of the composites was calculated via Equation (2).

(2) λ(W/m·K)=α×ρ×c

where, *α* is the thermal diffusivity (m^2^/s), *ρ* is the density (kg/m^3^), and *c* is the specific heat (J/kg·K).

#### 2.3.3. Mechanical Properties

Tensile and flexural tests were performed on a Universal Testing Machine (RGT-20A, Shenzhen Reger Instrument Co., Ltd., Shenzhen, China) at room temperature, according to ASTM D638 and ASTM D790, respectively. The impact tests of the composites were tested on an Impact Tester (XJ-50G, Hebei Chengde Mechanical Testing Machine Co., Ltd., Chengde, China), according to the Chinese Standards GB/T 1043. Each test was repeated at least eight times and the average values were reported.

#### 2.3.4. Scanning Electron Microscopy (SEM)

Samples were observed by a JSM-7500FSEM (FEI QUANTA 200, FEI Company, Eindhoven, The Netherlands) at an accelerating voltage of 12.5 kV. Surfaces of samples fractured in the liquid nitrogen were then gold-sprayed before the observation.

#### 2.3.5. Dynamic Mechanical Analysis (DMA)

The dynamic mechanical properties were performed on a DMA Q800 (TA Instruments, New Castle, DE, USA) with a single cantilever mode. The size of rectangular samples was 35 × 12 × 3 mm^3^ (L × W × H). The tests were carried out over a temperature range of −60 °C to 110 °C, with a heating rate of 3 °C ·min^−1^, at a frequency of 1 Hz.

#### 2.3.6. Rheology Properties

The rheological measurements were conducted on an AR2000ex Rotational Rheometer (TA Instruments, New Castle, DE, USA) using a wafer with the diameter of 25 mm. The dynamic strain sweep and frequency sweep were scanning at a strain range from 0.001% to 300% and a frequency range of 628.3–0.06283 rad·s^−1^, respectively.

#### 2.3.7. Differential Scanning Calorimetry (DSC)

DSC analysis was completed on a DSC Q20 (TA Instruments, New Castle, DE, USA). The samples were firstly heated from −20 to 200 °C at a rate of 10 °C·min^−1^ and kept at 200 °C for 5 min, and then cooled to −20 °C at a cooling rate of 10 °C·min^−1^, and remained at −20 °C for 1 min. Secondly, the samples were heated to 200 °C at the same heating rate, and finally stabilized at 200 °C for 1 min under the nitrogen atmosphere. The crystallinity (*X_c_*) of the composites can be calculated by Equation (3):

(3) $$X_{c}\left(\%\right)=\frac{\Delta H_{m}}{\varphi_{PLA}\times \Delta H_{m}^{0}}$$

where, Δ*H_m_* is the enthalpy of fusion (J/g), *φ_PLA_* is the actual weight fraction of PLA in the composites, and ${\Delta H}_{m}^{0}$ is the standard enthalpy of PLA for 100% crystallinity, equal to 93.7 J/g.

#### 2.3.8. Thermalgravimetric Analysis (TGA)

The thermal stability of the composites was characterized by a Q50 Thermalgravimetric Analyzer (TA Instruments, New Castle, DE, USA) from 25 to 600 °C, at a heating rate of 10 °C·min^−1^. The tests were carried out under nitrogen atmosphere with a nitrogen flow rate of 60 mL·min^−1^ and 40 mL·min^−1^ for samples and balance, respectively.

#### 2.3.9. Four Transform Infrared Spectroscopy (FTIR)

The FTIR spectra of the samples were recorded from 400 cm^−1^ to 4000 cm^−1^ with a Four Transform Infrared Spectroscopy (Nicolet 6700, Thermo Fisher Scientific, Waltham, MA, USA). The powder of graphite and TA-G were pressed with KBr for the measurement.

## 3. Results and Discussion

### 3.1. Effect of Graphite Content on PLA/G Composites

#### 3.1.1. Electrical Resistivity

**Figure 2:**
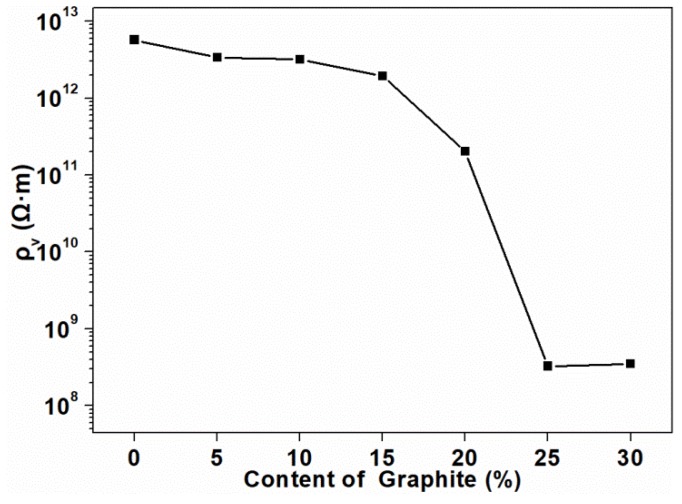
Volume electrical resistivity of the composites at different loadings of graphite.

Figure 2 shows the volume electrical resistivity of the composites as a function of the graphite content. Three replicates were tested in each group to obtain the average value, and the standard deviation was calculated, which was equal to 0. The volume electrical resistivity of the composite without graphite was calculated to be in the order of 10^12^ Ω·m. It was clearly shown that, with the increase of graphite content, the volume electrical resistivity of the composites gradually decreased, but the order of magnitude did not change. When the added amount exceeded 15 pbw, the volume resistivity of the composites significantly decreased, which meant that the conductive paths in the composite began to form and the graphite began to contact with each other. Until the content reached 25 pbw, the magnitude of the resistivity reached the lowest value (10^8^ Ω·m), which indicated that the conductive path in the composite was completely formed at this time. The volume electrical resistivity of the composites dropped significantly between 15 pbw and 25 pbw, and at which point the electrical conduction path formed by graphite in the PLA matrix reached the percolation threshold [36]. As graphite continued to be added into the composites, the resistivity no longer changed in magnitude. This phenomenon indicated that the percolation threshold for the electrical conductivity of the composites was lower than 25 pbw. The formation of the percolation threshold was mainly attributed to the better conductive network constituted by graphite. The contact resistance also existed in the percolating network formed within the composites [37].

#### 3.1.2. Thermal Conductivity Results

**Figure 3:**
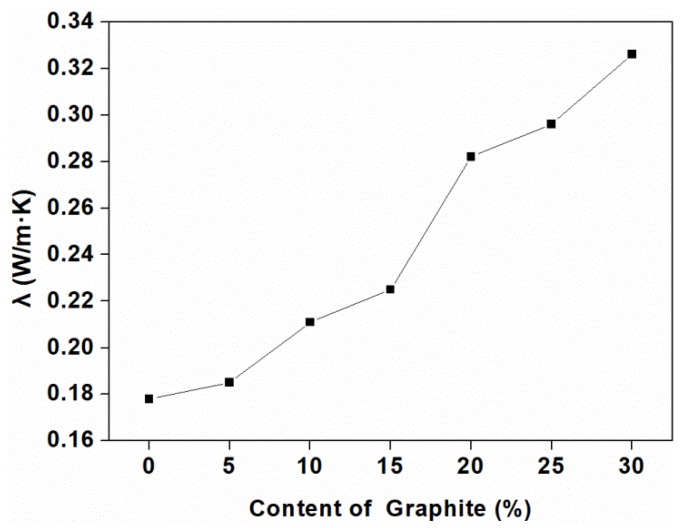
Thermal conductivity of the composites at different loadings of graphite.

**Figure 4:**
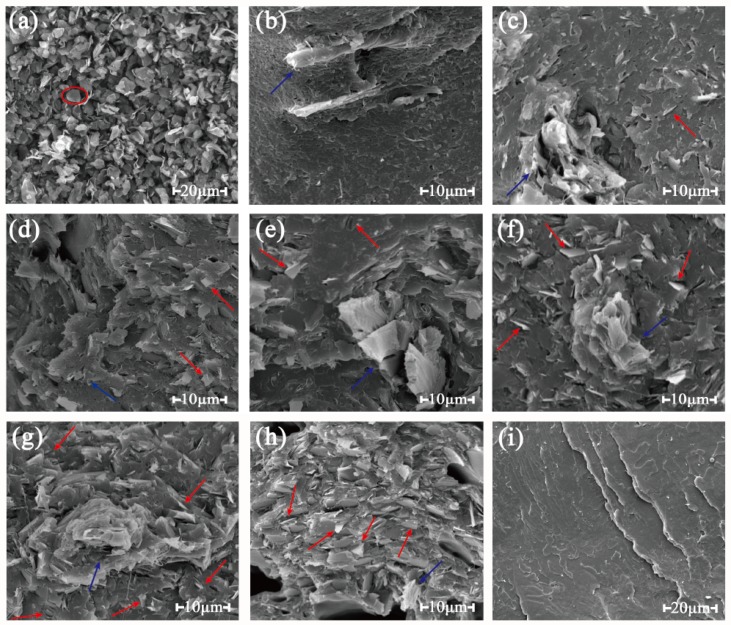
SEM micrographs of the fracture surfaces of composites with different graphite content: (**a**) neat graphite, (**b**) 0 pbw graphite added in the composite, (**c**) 5 pbw, (**d**) 10 pbw, (**e**) 15 pbw, (**f**) 20 pbw, (**g**) 25 pbw, (**h**) 30 pbw, (**i**) pure polylactic acid (PLA).

Figure 3 shows the thermal conductivity of the composites as a function of the graphite content. Three samples were tested and averaged of each group, and the standard deviation was calculated to be very small, so it could not be reflected on the graph. It can be seen that the thermal conductivity increased at higher graphite content. This was shown that the transport path required for high thermal conductivity was greatly improved by the average carbon free path and the conductive network produced by adding graphite [37], and the fact can be explained by the morphology of the composites showed in Figure 4.

#### 3.1.3. Morphology

It can be seen in Figure 4 that the blue arrow pointed to the wood flour, and the red arrow stood for the graphite dispersed in the matrix. The neat graphite appeared like a sheet-like flake (for example in the red cycle of Figure 4a). It can be seen in Figure 4i that the fracture surface of pure PLA was smooth, meaning that a typical brittle fracture was obtained. In Figure 4b–h, it can be seen that graphite flakes disperse randomly in the polymer matrix [38]. With the increase of the graphite content, the dense conductivity network was formed and the graphite flakes contacted each other, which meant the formation of the conductive paths.

#### 3.1.4. Mechanical Properties

**Figure 5:**
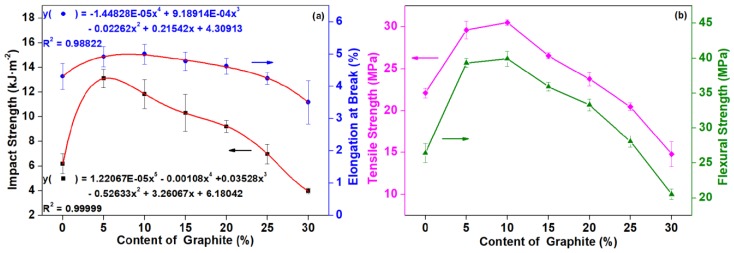
Mechanical properties of PLA/wood flour (WF)/nano-graphite (G) composites as affected by different content of graphite: (**a**) impact strength and elongation at break; (**b**) tensile strength and flexural strength.

The impact strength and elongation at break of composites with different contents of graphite are shown in Figure 5. With the addition of graphite, the impact strength firstly increased and then decreased. With the loading content of 5 pbw, the impact strength of the composite was the highest, reaching 13.10 kJ·m^−2^, with an increase of 111.97%. As the graphite content continually increased, the impact strength of the composite showed a decreased trend. However, when the amount of graphite added was 25 pbw, the impact strength of the composite was still higher than that of the composite without graphite. When the addition amount reached 30 pbw, the impact strength was lower than that of the composite material without graphite powder. By adding 30 pbw graphite, the impact strength of the composite was the lowest, reaching 3.97 kJ·m^−2^, which was 35.76% lower than that of the composite without graphite. The relationship between impact strength, elongation at break, and graphite can be obtained by a nonlinear curve-fitting (the red line in Figure 5a).

As can be seen in Figure 5, the tensile strength and elongation at break of the composites enhanced with the graphite addition of 5–20 pbw and reached a maximum with 10 pbw graphite loading. By continually increasing the graphite content to 25 pbw, the tensile strength and elongation at break of the composites decreased slightly, but when the added amount reached 30 pbw, the tensile properties were significantly reduced. When 0–25 pbw graphite was added, the flexural strength of the composites was increased, and the tendency was firstly increased and then decreased. The flexural strength initially increased to be 39.33 MPa at the 5 pbw graphite content, continued to rise to the maximum of 39.93 MPa with the graphite content of 10 pbw, and decreased at higher graphite contents. The flexural strength of the composite reduced by 22.19% until the graphite addition amount was 30 pbw.

In summary, it was a critical value when the amount of graphite added in the composite was 25 pbw, according to both mechanical properties and conductivities. At this time, the impact strength and flexural strength of the composites were increased compared with the composite without graphite, which were increased by 12.78% and 6.32%, respectively. The elongation at break remained essentially unchanged, and the tensile strength decreased slightly (7.33%). With the addition of graphite, the mechanical strength of the composites increased, which may be due to the reinforcing effect of the uniform dispersion of graphite in the matrix [36]. As the graphite content was further increased, it would agglomerate, resulting in the poor dispersion and stress concentration [37]. When excess graphite was added, the continuity of the entire matrix changed. Due to the further increase of graphite content, the agglomeration was more likely to occur, which may cause an adverse effect of the deformation of the matrix, and energy transfer or diffusion under stress, but mainly caused the stress concentration in the matrix, so that the toughness of the composite reduced and the brittleness increased [39]. Luo et al. also reported the similar results. With the addition of carbon nano-fillers, the elongation at break and tensile strength of PLA/carbon nano-fillers composites increased firstly and then decreased. When the content of carbon nano-fillers was less than 5 wt %, the elongation at break and tensile strength of PLA/carbon nano-fillers composites could be improved. When the amount reached 7 wt %, the tensile properties of the composite were adversely affected [40].

#### 3.1.5. DMA Results

**Figure 6:**
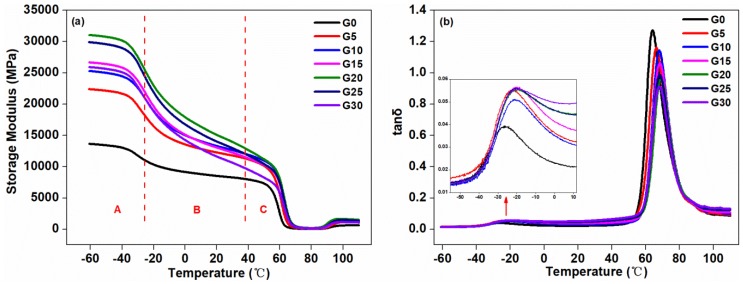
Dynamic mechanical analysis (DMA) curves of the composites with different graphite content: (**a**) temperature dependence of storage modulus (*E*’), (**b**) loss factor (tanδ) versus temperature.

The compatibility and the glass transition temperature (*T*_g_) were often examined by DMA measurements [41]. Figure 6 shows the plot of the storage modulus and loss factor of the composites as a function of temperature. As shown in Figure 6a, the storage modulus of the composite material can be characterized by three regions, i.e., Regions A, B, and C. In Region A, the storage moduli of the graphite-added composites were higher than that of the control composite without graphite, which indicated that the rigidity of the composites was increased after the addition of graphite. This was because of the presence of the dispersed graphite, which was a kind of stiff filler. Moreover, the chain mobility of the composite was limited in this region, so the storage modulus of the composites in Region A was the largest among the three regions. As the graphite content increased, the storage moduli of the composites increased, and when the addition amount was 25 pbw, the storage modulus was the largest. Continually increasing graphite to 30 pbw, the storage modulus dropped suddenly. In Region B, the storage moduli of the graphite-added composites were still higher than the control. With the temperature increasing, the storage modulus suddenly reduced, which may be due to the aggregation of the graphite [42]. The composite with the graphite addition of 30 pbw had the largest reduction in storage modulus, and this sudden drop suggested a high degree of aggregation of graphite dispersed in the matrix. In Region C, as the temperature increased, the storage moduli of the composites dropped sharply, which was attributed to the fact that the frozen segmental motion of the composites began to be released as the temperature increased to around the glass transition temperature. When the temperature was higher than the glass transition temperature and continually increased to 70 °C, the movement of the composite segment was free, and the storage modulus of the composites remained substantially unchanged.

It can be seen from Figure 6b that the loss factor can be used to measure the characteristic temperature peaks matched with relaxation processes. Two relaxation peaks can be seen in the plot, around −26.39 and 63.88 °C, which represented the phase separation of the composite at the microscopic level. This corresponded to the glass transition temperature of TPU and PLA, respectively, due to the movement of molecular segments in the amorphous region of the two components. As shown in Table 3, the addition of graphite had an influence on the miscibility of the composites. Due to the interaction between the two components, the TPU and PLA were partially miscible [43]. Furthermore, with the increase of graphite content, the glass transition temperature of the component PLA moved to a higher temperature until the added amount reached 15 pbw, and the glass transition temperature of TPU gradually approached the glass transition temperature of the PLA. Therefore, there existed a relatively low value of Δ*T*_g_, suggesting an increase in miscibility between the components of the composite. By continually increased graphite content to 20 pbw, 25 pbw, or 30 pbw, the Δ*T*_g_ values of the composites began to increase, and the glass transition temperature of the component TPU began to shift away from the glass transition temperature of the composites, which represented a decrease in miscibility. This was consistent with the results of mechanical performance and supported the observation of the stiffness effect of graphite in the surface morphology of the composites.

#### 3.1.6. Rheological Properties

**Figure 7:**
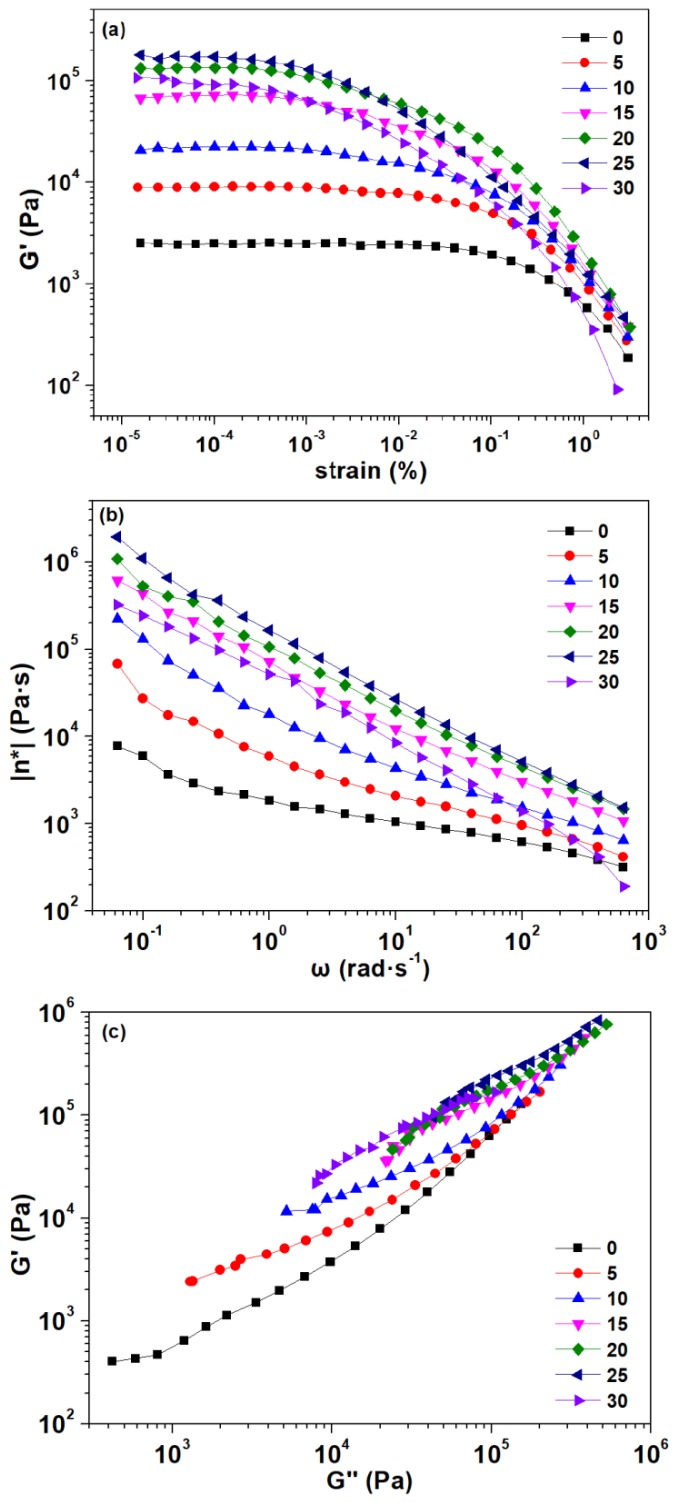
Plots of (**a**) storage modulus (*G*′) as a function of strain, (**b**) complex viscosity (η^*^) as a function of frequency, (**c**) storage modulus (*G*′) versus loss modulus (*G*″) in dynamic frequency sweep of the composites.

The strain sweep was used to study the effect of graphite addition on the rheological properties of the composites and to determine the linear viscoelastic region of the composites to ensure that the other tests were carried out within the linear viscoelastic region. The storage modulus as a function of the strain is shown in Figure 7a. The control composite exhibited a typical strain softening behavior, which meant that the TPU particles were uniformly dispersed in the PLA. With the addition of graphite, the storage moduli of the composites were firstly increased and then decreased, but always larger than the control composite. Furthermore, the strain softening phenomenon became more obvious, especially when the amount of graphite added reached 25 pbw or 30 pbw. This was suggested that an excessive amount of graphite caused an agglomeration of the graphite, making it impossible to homogeneously disperse in the composites.

The complex viscosity as a function of frequency can be observed in Figure 7b. All composites showed a shear thinning behavior, which was the typical characteristic of non-Newtonian fluids. As the graphite content increased, the complex viscosity of the composite increased, because more solid fillers added into the composites may require a greater shearing rate to achieve the critical shear flow [44]. The shear thinning behavior was enhanced, due to the more solid-like behavior [45]. However, when the addition amount reached 30 pbw, the complex viscosity of the composite suddenly decreased, which may be due to the excessive graphite addition leading to agglomeration and making the filler particles larger. Therefore, the possible network formed by the graphite might be a reason for the solid-like response of the graphite-added composites [42]. This is consistent with the composites’ morphology observed previously.

The Han plot of storage modulus versus the loss modulus shown in Figure 7c can be used to evaluate the miscibility of the composites [46]. The homogeneity and compatibility of multiphase systems can be reflected by the linear correlation in the plot. The miscible system can observe the same slope as the control composite in the plot. Similarly, a different slope observed in the Han plot may be an indication of the immiscibility or phase separation of the multiphase system [41]. As can be seen from the Han plot, all the curves exhibited nonlinear correlations. Moreover, the slope was smaller in the region where the loss modulus was low. The slope of the composites at lower graphite content (5 pbw and 10 pbw) were similar to that of the control composite, indicating the similar compatibility of the three. When increasing the graphite content, the correlation deviation of the composites in the Han plot can be observed more obviously, which indicated that the compatibility of the composites became worse.

#### 3.1.7. Thermal Stability

**Figure 8:**
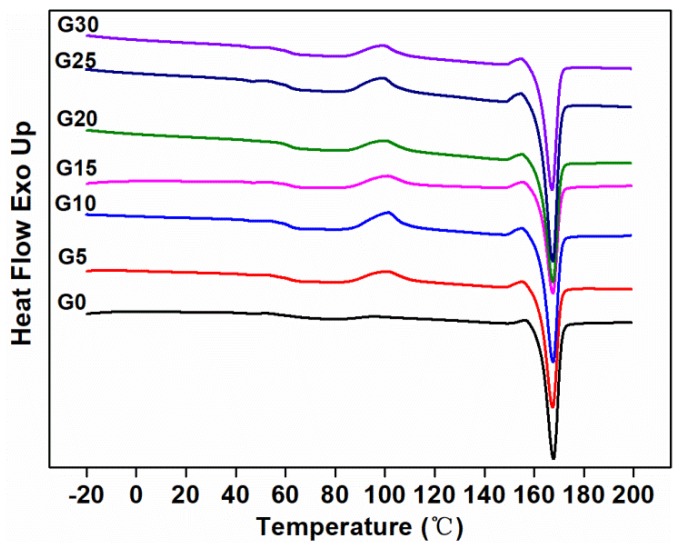
Second melting DSC curves of composites.

The second heating curves of the composites measured by DSC are shown in Figure 8, and the characteristic values of the curves are shown in Table 4. It can be seen that the cold crystallization of the composite without graphite occurred at 95.88 °C, followed by a melting peak at 167.70 °C. Since the TPU component in the matrix was amorphous, the crystallization behavior of the composites was attributed to the PLA component. With the addition of graphite, the cold crystallization temperature of the composites increased. This may be due to the addition of a certain amount of graphite, which could restrict the motion of the PLA chains, thereby limiting the cold crystallization process of the PLA, resulting in an increase in the cold crystallization temperature of the PLA in the composites [47,48]. With the increasing loading of graphite in the composites, no significant changes were observed in the fusion points. The crystallinity of G25 was almost the same as G0, which was consistent with the mechanical properties.

**Figure 9:**
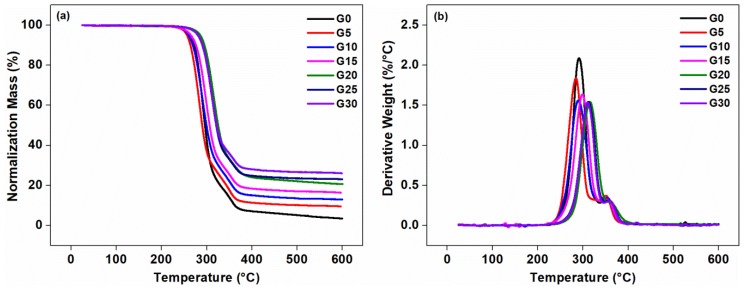
Thermalgravimetric analysis (TGA) curves (**a**) and derivative thermalgravimetric analysis (DTG) curves (**b**) of the composites with varying graphite content.

The thermal stabilities of the composites were obtained from TGA, as shown in Figure 9 and Table 4. For the composites with or without graphite, a two-step decomposition process can be seen in Figure 9. The initial decomposition probably occurred at about 300 °C, which represented the decomposition of PLA in the matrix, while the next decomposition process at about 350 °C meant the decomposition of TPU in the matrix.

The onset decomposed temperature of G0 was higher than that of the G5, and that was because the thermal conductivity of graphite was high, resulting in rapid diffusion of heat in the matrix. The transfer of temperature and heat required a time process. The addition of graphite allowed the matrix to transfer energy well before decomposition, thus shortening the entire process. The thermal conductivity of the matrix was poor, which delayed the thermal degradation time. According to the heating rate of 10 °C·min^−1^ of the thermogravimetric analysis, the onset decomposed temperature of G0 was delayed by about 1 min compared with G5. With the further increase in graphite content (G20–G30), the onset decomposed temperature of the composites became higher than G0, which may be due to the un-homogeneous dispersion of too much graphite in the matrix, which caused the thermal conductivity to be hindered. The residue of the composites at the printing temperature (210 °C) was still about 99.50 wt %, and the thermal weight loss was small, which indicated that the composite cannot undergo serious degradation and damage during FDM printing. This was further suggested that 210 °C was a suitable temperature for FDM technology. The residue of the composites at 600 increased with the increasing content of the graphite, indicating that the addition of the graphite into the composites can improve the thermal stability of the composites [47,49,50,51].

### 3.2. Properties of the Modified Composites

To improve the conductivity of the composites, TA-G was prepared as a functionalized conductivity filler in the composites. The rGO and MCNT were added into the composites for the same purpose.

#### 3.2.1. FTIR Results

**Figure 10:**
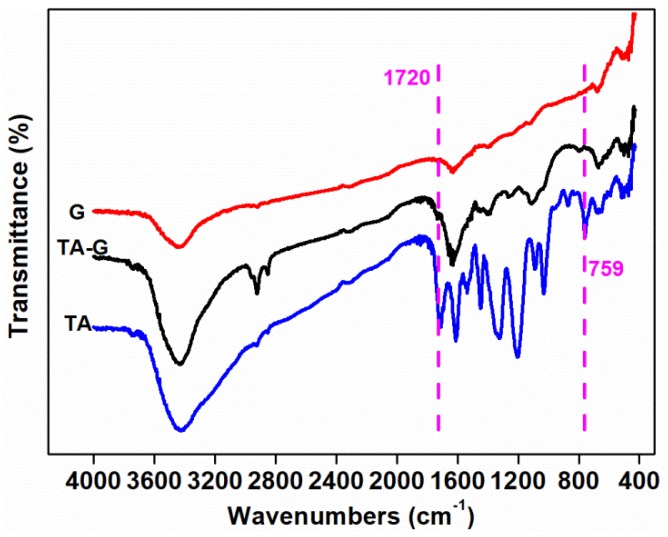
FTIR spectrum of tannic acid (TA), nano-graphite (G), and TA-G.

Figure 10 shows the FTIR spectrum of TA, G, and TA-G. For G, the characteristic absorption peaks at 3423, 1630, and 674 cm^−1^ represented the stretching vibration of O–H, the stretching vibration of C=C group, and the bending vibration of C–H, relatively. Compared with G, new characteristic absorption peaks of TA-G at 1720 cm^−1^ and 795 cm^−1^ were related to the stretching vibration of –C=O group and the bending vibration of C–H on the benzene. Some of the characteristic absorption peaks enhanced, which were at 2924, 2854, 1630, 1112 cm^−1^, indicating the bending vibration of –CH_3_, –CH_2_, the stretching vibration of C=C, and the =C–O–C group. The enhancement in intensities was due to the introduction of TA on the one hand and the exfoliation of a few graphene layers from graphite during the preparation process on the other hand. The results showed that the characteristic peaks of TA can be observed also at the TA-G, which suggested that TA was successfully functionalized G by the π–π conjugative effect [52,53].

#### 3.2.2. SEM Images and EDX Spectrum

**Figure 11:**
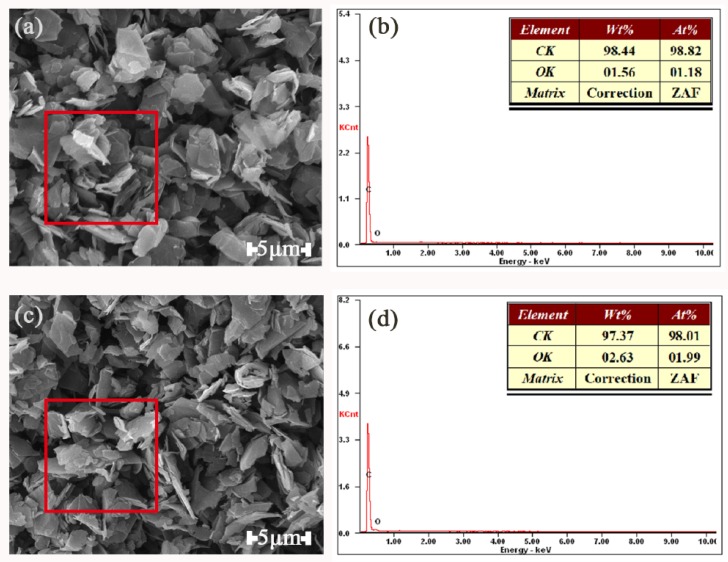
SEM Photographs of G (**a**) and TA-G (**b**) and EDX spectrum of G (**c**) and TA-G (**d**).

**Figure 12:**
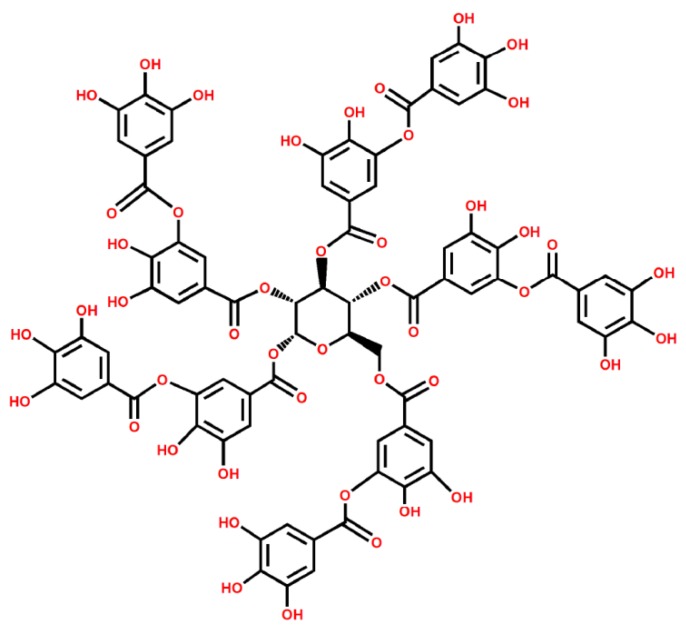
Structural formula of TA.

Figure 11 shows EDX spectrum and SEM images of G and TA-G. The red square represents the area of the X-ray scan spectrum. The data in the table represented the relative mass fraction and atomic percentage of carbon and oxygen in the graphite before and after the modification, respectively. The molecular formula of TA is C_76_H_52_O_46_ and the structural formula is shown in Figure 12. The atomic percentage of the oxygen atoms in TA-G (1.99%) was obviously higher than that in G (1.18%), which further proved that the molecules of TA were successfully adsorbed onto G.

#### 3.2.3. Conductivities of the Modified Composites

To improve the conductivity of the composites, TA-G, rGO, and MCNT were used to combine with G.

**Figure 13:**
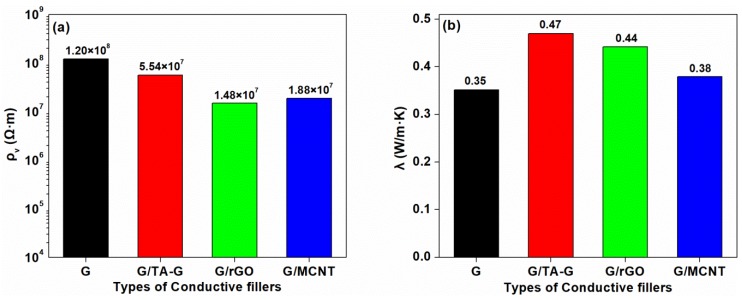
Electrical Resistivity (**a**) and Thermal Conductivity (**b**) of the Composites.

The electrical resistivity and the thermal conductivity are shown in Figure 13. The addition of TA-G slightly reduced the electrical resistivity of the composite, and the thermal conductivity increased to 0.47 W/m·K. This may be due to the better-dispersed of TA-G in the matrix, improving the interfacial compatibility. The enhanced interfacial affinity between G and the matrix can decrease the interfacial thermal resistance and phonon scattering, resulting in the increasing thermal conductivity. The best effect was the combination of G and rGO, which reduced the electrical resistivity of the composite by an order of magnitude and increased the thermal conductivity by 25.71%. With the addition of MCNT, the resistivity of the composite reduced by 84.33%, which, however, did not reach an order of magnitude, and the thermal conductivity was hardly improved.

#### 3.2.4. Models of PLA/G-rGO Composites by FDM

**Figure 14:**
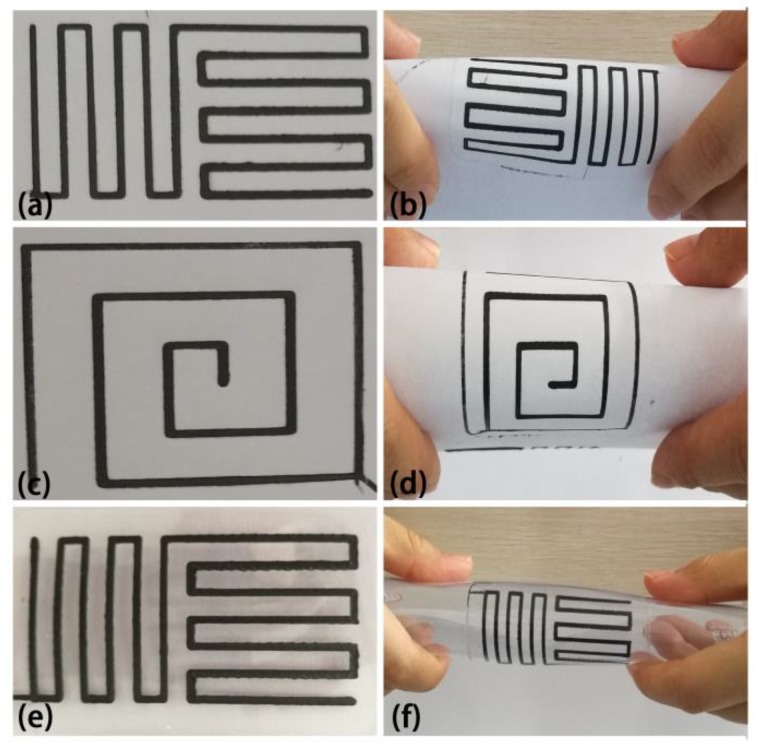
Models printed by FDM on different baseplates: different shapes printed on the paper (**a**,**c**), and their bend parts (**b**,**d**); models printed on the flexible materials (**e**), and its bend parts (**f**).

The PLA/G-rGO composite was chosen to print the models by FDM, due to the better electrical resistivity and the thermal conductivity. As shown in Figure 14, the single layer models were printed on different baseplates, such as (a) and (c) on the paper, (e) on the flexible materials. The combination of the printing filaments and the baseplate were better, and by bending the baseplate, it can be seen that the printed model had a certain flexibility and cannot be separated from the baseplate when the baseplate was bent.

## 4. Conclusions

In order to improve the electrical and thermal conductivity, PLA-based biocomposites were fabricated by FDM using different conductive fillers, such as nano-graphite, TA-G, rGO, and MCNT. The following conclusions were drawn from this study: (1) It was found that the volume resistivity of the composites decreased significantly, reaching 10^8^ Ω·m with the addition amount of 25 pbw graphite. The thermal conductivity, mechanical properties, complex viscosity and the moduli were proved to be increased using this graphite content compared to other contents. (2) DSC results showed that, with the addition of graphite, the cold crystallization process of the PLA was limited, resulting in an increase in the cold crystallization temperature. The results of TGA suggested that the thermal stability of the composites was improved and 210 °C could be a suitable temperature for FDM technology. (3) TA-G was characterized and proved to be successfully prepared. (4) The combination of TA-G, rGO, and MCNT with graphite could further improve the electrical and thermal conductivity of the composites. The results showed that combined rGO with graphite can be an effective way, because it can reduce the resistivity by an order of magnitude and increase the thermal conductivity by 25.71%. (5) Models printed by FDM illustrated that the composite filaments had a certain flexibility and can be printed onto paper or flexible baseplates.

## Figures and Tables

**Table 1:** Formulation of the composites with different contents of graphite.

Sample Codes	PLA/pbw ^1^	TPU/pbw	WF/pbw	PEWax/pbw	G/pbw
G0	70	20	10	0.5	-
G5	70	20	10	0.5	5
G10	70	20	10	0.5	10
G15	70	20	10	0.5	15
G20	70	20	10	0.5	20
G25	70	20	10	0.5	25

^1^ pbw = part by weight.

**Table 2:** Formulation of the modified composites.

Sample Codes	PLA/pbw	TPU/pbw	WF/pbw	PEWax/pbw	G/pbw	TA-G/pbw	rGO/pbw	MCNT/pbw
G	70	20	10	0.50	25	-	-	-
G/TA-G	70	20	10	0.50	12.50	12.50	-	-
G/rGO	70	20	10	0.50	18.75	-	6.25	-
G/MCNT	70	20	10	0.50	18.75	-	-	6.25

**Table 3:** *T*_g_ Values of the composites with different graphite content.

G/pbw	*T*_g(TPU)_/°C	*T*_g(PLA)_/°C	Δ*T*_g_(*T*_g(PLA)_ − *T*_g(TPU)_)
0	−26.39	63.88	90.27
5	−23.32	66.28	89.60
10	−21.01	68.17	89.18
15	−20.55	68.40	88.95
20	−21.52	68.32	89.84
25	−22.17	68.22	90.39
30	−22.84	67.98	90.82

**Table 4:** Characteristic values of the DSC and TGA curves of the composites with different graphite content.

Sample Codes	*T*_m_^2^/°C	*T*_cc_^3^/°C	*X*_c_^4^/%	*T*_onset_^5^/°C	*T*_max_^6^/°C		Residue at 210/wt %	Residue at 600/wt %
					*T*_PLA_	*T*_TPU_		
G0	167.7	95.88	46.97	272.19	291.09	355.27	99.41	3.46
G5	167.41	100.88	44.35	262.67	284.43	350.68	99.35	9.48
G10	167.56	101.28	43.06	267.21	289.76	352.53	99.43	12.94
G15	167.5	100.95	40.24	276.05	299.03	352.97	99.45	16.35
G20	167.54	100.21	46.42	292.46	315.28	362.27	99.6	20.65
G25	167.45	98.89	47.07	288.22	311.86	360.05	99.67	23.06
G30	167.2	99.28	49.07	288.13	310.6	359.61	99.69	26.14

^2^*T*_m_ represents the fusion point of the composites, ^3^ *T*_cc_ represents the cold crystallization temperature, ^4^ *X*c represents the crystallinity, ^5^ *T*_onset_ represents the epitaxial onset temperature, ^6^ *T*_max_ represents the temperature at which the maximum weight loss rate occurred.
